# Controlled Microwave Heating Accelerates Rolling Circle Amplification

**DOI:** 10.1371/journal.pone.0136532

**Published:** 2015-09-08

**Authors:** Takeo Yoshimura, Takamasa Suzuki, Shigeru Mineki, Shokichi Ohuchi

**Affiliations:** 1 Department of Applied Biological Science, Tokyo University of Science, 264 Yamazaki, Noda, Chiba 305–8506, Japan; 2 Graduate School of Life Science and System Engineering, Kyushu Institute of Technology, 680–4 Kawazu, Iizuka, Fukuoka 820–8502, Japan; New England Biolabs, Inc., UNITED STATES

## Abstract

Rolling circle amplification (RCA) generates single-stranded DNAs or RNA, and the diverse applications of this isothermal technique range from the sensitive detection of nucleic acids to analysis of single nucleotide polymorphisms. Microwave chemistry is widely applied to increase reaction rate as well as product yield and purity. The objectives of the present research were to apply microwave heating to RCA and indicate factors that contribute to the microwave selective heating effect. The microwave reaction temperature was strictly controlled using a microwave applicator optimized for enzymatic-scale reactions. Here, we showed that microwave-assisted RCA reactions catalyzed by either of the four thermostable DNA polymerases were accelerated over 4-folds compared with conventional RCA. Furthermore, the temperatures of the individual buffer components were specifically influenced by microwave heating. We concluded that microwave heating accelerated isothermal RCA of DNA because of the differential heating mechanisms of microwaves on the temperatures of reaction components, although the overall reaction temperatures were the same.

## Introduction

**Fig 1:**
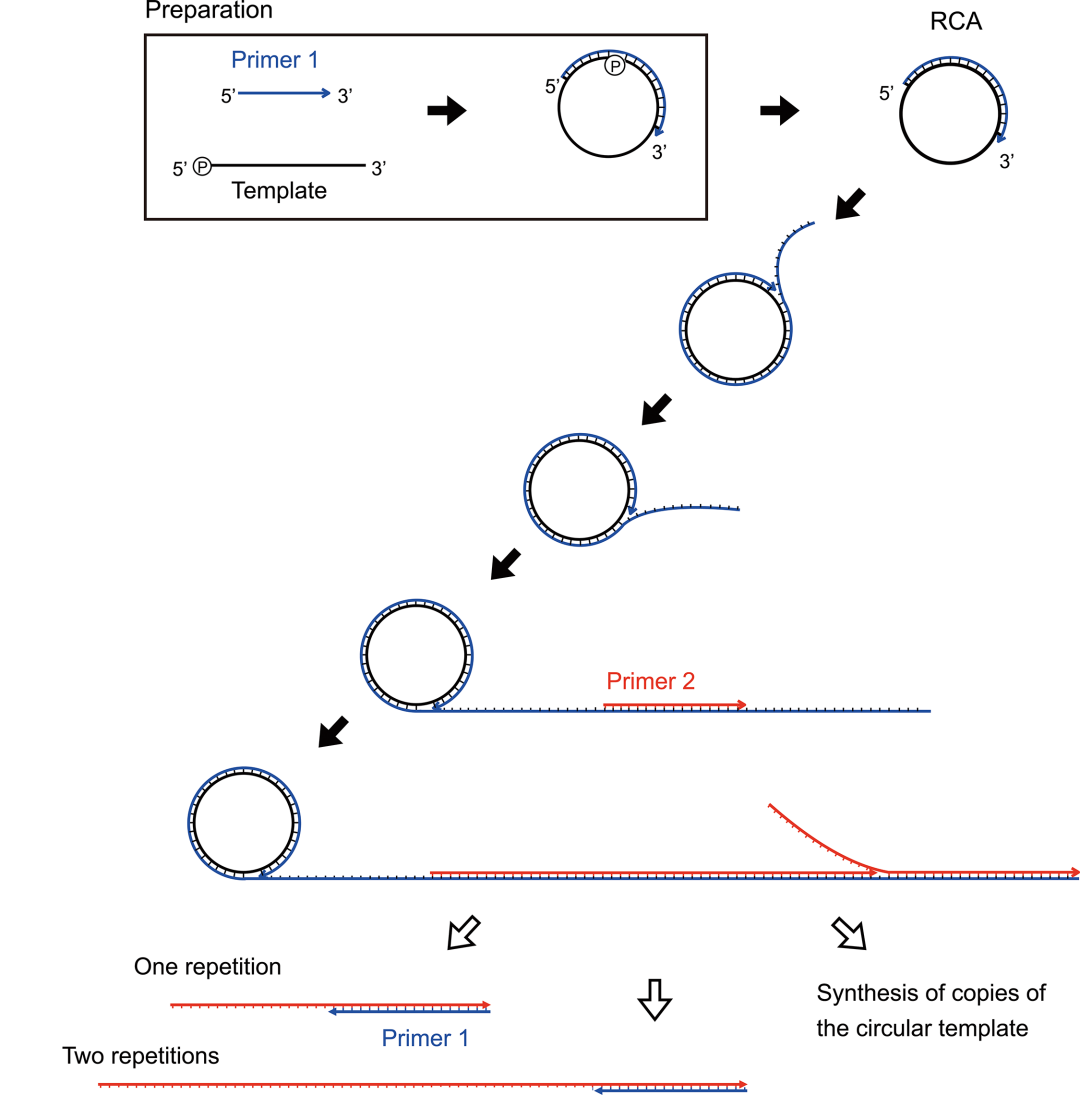
Rolling circle amplification (RCA) showing the circularized template and two primers. Primer 1 initiates the RCA reaction, and reverse primer 2 anneals to each tandem repeat generated by the rolling circle.

Rolling circle amplification (RCA), which is based on the mechanism of the replication of viral genomes, is an isothermal nucleic acid amplification technique that uses two primers, a circularized template and a DNA polymerase with strand displacement activity [1–5]. RCA efficiently synthesizes many copies of repeated sequences from circular DNA and RNA templates (Fig 1). Moreover, it detects single nucleotide polymorphisms using a circularized DNA probe called a *padlock probe* as well as efficiently amplifying bacterial genomes by a hyper-branch method [6–10]. Isothermal DNA polymerization has various application possibilities [11–14]. Microwave heating technology is applied to organic and inorganic chemistry using a microwave generator to produce useful effects such as rapid heating, decreased reaction times, and improved product yield and purity[15–18]. The microwave effect is debated to be the “Thermal-effect,” “Non-thermal effect,” and “Specific microwave effect (superheating or selectivity heating)”[19, 20]. One of the mechanisms of microwave heating in organic chemistry involves heating a low molecular weight compound with a dipole polarization and an ionic conduction. Enzymatic reactions (in the fields of proteomics, pretreatment, degradation, chemical synthesis, and optical resolution) are performed using microwave heating [21–25]. Published studies on PCR using microwave heating (microwave-assisted PCR, MW-PCR) show that amplicon synthesis is inefficient, large reaction volumes are required, and temperature is difficult to control or measure [26]. We reasoned that these problems may be attributed to the use of three temperatures (for annealing, elongation, and denaturation) in the MW-PRC method that are not precisely controlled by microwave heating. Temperature measurement under microwave irradiation is a matter of importance [27, 28]. For strict temperature control by microwave irradiation, a gene amplification study using microwave was reported in Milliliter-scale PCR [29]. Microwave device with several frequencies for PCR is developed to accurately control temperature [30, 31]. In contrast, RCA does not require complex temperature control compared with conventional PCR methods, and isothermal conditions are appropriate for controlling microwave heating. In our previous study, we reported the microwave-assisted RCA for the first time using a multi-mode microwave generator[32], but the reproducibility was difficult. We developed a novel single mode resonant cavity microwave generator with fiber-optic probe to improve accurate temperature measurement and reproducibility. Further, this applicator can be used by a conventional PCR-scale (50–100 μL). MW-RCA was performed using a circular DNA template and four thermostable DNA polymerases with strand displacement activity. We focused on microwave selectivity heating, which is one of the features of microwave heating. Microwave selectivity heating is thought to alter molecular motion state compared with conventional heating when the reaction temperature is the same. To examine the components of RCA responsible for microwave selectivity heating, we measured the temperature of RCA components under conventional and microwave heating conditions at the same temperature. MW-RCA, which increased the components of selectivity heating, was performed.

Here we show that microwave heating facilitated the synthesis of repetitive DNA through RCA using the four DNA polymerases. Analysis of the temperature profiles of each RCA component subjected to microwave heating revealed the selectivity heating of buffer components compared with primers, template DNA, dNTP, and RNase-free water. We show further that the components of RCA responsible for microwave selectivity heating related to the acceleration of MW-RCA.

## Materials and Methods

### Primers and templates

The RCA template and primers were designed to amplify sequences encoding a zinc finger protein for the expression of an artificial repetitive protein. The terminal region of primers contained restriction enzyme sites of *Bam*HI and *Hind*III. Template (75 nucleotides [nt]): 5′-CTGTGCAAACACTACATTTGCTCTTTTGCCGACTGTGGCGCTGCTTATAACAAGAACTGGAAACTGCAGGCGCAT-3′. Primer-1s (45 nt): 5′-GGATCCTACATTTGCTCTTTTGCCGACTGTGGCGCTGCTTATAAC-3′. Primer-2 (44 nt): 5′-AAGCTTTAGTGTTTGCACAGATGCGCCTGCAGTTTCCAGTTCTT-3′.

### RCA reactions, circularization

A circular RCA template was prepared according to previous published study [32]. Template oligonucleotide (0.1 nmol) was phosphorylated using T4 polynucleotide kinase (10 U) in 100 μL of solution at 37°C for 60 min. After heating at 72°C for 10 min, the phosphorylated template nucleotide (1 μL) was diluted with 36 μL of LK buffer (50 mM Tris–HCl, 10 mM MgCl_2_, 10 mM dithiothreitol, 1 mM dATP, and 25 μg/mL bovine serum albumin) containing 0.1 nmol of primer (primer-2). The primer–template mixture was annealed and incubated at 16°C for 1 h with T4 DNA Ligase (1050 U) in 950 μL of LK buffer. The circularized primer–template was precipitated from the phenol–chloroform–isoamyl alcohol extraction mixture and suspended in RNase-free water (TaKaRa). The DNA concentration of the final sample used for RCA was determined using a NanoDrop 2000c (Thermo Scientific) and was diluted to a final concentration of 60 ng/μL.

### RCA

RCA reactions were performed to amplify the repetitive sequences using DNA polymerases with strand-displacement activity. The four thermostable DNA polymerases were as follows: *Bst* DNA polymerase Large Fragment (*Bst*-LF) (New England Biolabs, NEB), *Bst* DNA polymerase, *Csa* DNA polymerase, and 96–7 DNA polymerase (Nippon Gene). All RCA reactions were performed in a final volume of 50 μL at the optimum temperature of each enzyme in a 0.2-mL polypropylene tube (QSP) using a thermal-cycler (TaKaRa). Reactions were initiated after raising the temperature from 13°C to each initial temperature (60°C or 55°C) for 2 min. The control RCA reaction contained a dNTP mixture (each 2.5 mM), 10 pmol each of two primers, buffer specific for each DNA polymerase, eights units of each DNA polymerase, and 60 ng of each circular template–primer complex. *Bst*-LF was incubated in ThermoPol Buffer (20 mM Tris–HCl, 10 mM (NH_4_)_2_SO_4_, 10 mM KCl, 2 mM MgSO_4_, and 0.1% Triton X-100; NEB) at 60°C for 20–60 min. The *Bst* DNA polymerase (Nippon Gene) was incubated in the *Bst* reaction buffer (8 mM Mg^2+^) at 60°C for 20–60 min. The *Csa* DNA polymerase (Nippon Gene) was incubated in the *Csa* reaction buffer (8 mM Mg^2+^) at 60°C for 10–60 min. The 96–7 DNA polymerase (Nippon Gene) was incubated in the 96–7 reaction buffer (9.5 mM Mg_2_^+^) at 55°C for 10–180 min. A thermal-cycler was used to inactivate each DNA polymerase.

### Microwave applicator

**Fig 2:**
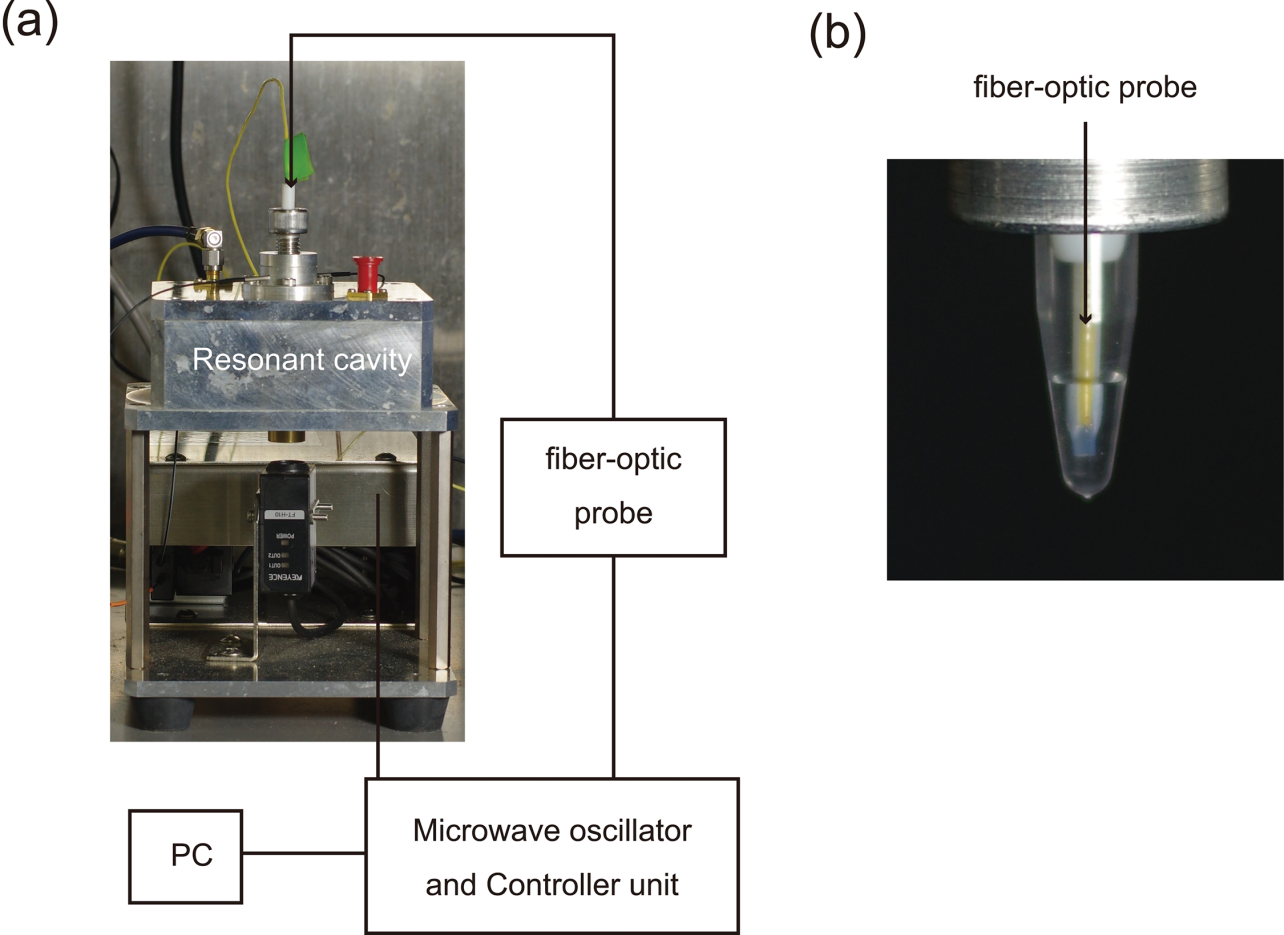
Resonant cavity-type microwave system (a) Resonant cavity of TM_010_ mode (b) polymerase chain reaction tube with a fiber-optic probe.

Microwave-assisted heating experiments were performed using a microwave applicator in TM_010_ mode (SAIDA FDS), equipped with a fiber-optic probe (Neoptix) for internal online temperature control (Fig 2). This resonant cavity-type microwave applicator (2.45 GHz) more precisely controls output compared with the magnetron power generated by a solid-state device (maximum 10 W). All microwave reactions were performed using a 0.2-mL polypropylene tube (QSP). This instrument was operated in a cold room (5°C) for the continuous generation of microwave power. This cavity resonator allows users to control the temperature of a 50 μL volume reaction mixture.

### MW-RCA

The temperature of the MW-RCA reaction mixture contained in a PCR tube was directly measured and was controlled using the fiber-optic probe (Fig 2B). All MW-RCAs were performed in a cold room. To prevent exceeding the target temperature, the temperature of the MW-RCA reaction mixture was raised from 13°C to 60°C in 2 min, and the reactions were incubated for 30 min (S1 and S5 Figs) because the microwave applicator was unable to maintain the reaction volume at 60°C over 30 min. The average microwave output at 60°C during the 30-min incubation was <2 W (S2 and S6 Figs). After terminating the MW-RCA reaction, DNA polymerases that were used in microwave heating were denatured by placing the tubes in a heating block at 90°C.

### Temperature measurements of RCA and buffer components

Conventional heating using a 60°C heating block and microwave heating were measured using a fiber-optic probe in the cold room for 10 min beginning at 13°C. The data used to plot the temperatures represent the average of three measurements.

### Preparation of RCA mixtures containing a 4-fold increased concentration of each of RCA component

Three RCA mixtures each containing a 4-fold excess concentration of the template–primer complex (200 ng) with two primers (each 40 pmol), dNTP (each 10 mM), and *Bst*-LF (32 U) were made up to 50 μL using RNase-free water. RCA mixtures each containing one component in 4-fold excess of its standard concentration in ThermoPol Buffer (NEB) were prepared as follows: 1 μL of each component (3 M Tris–HCl [pH 8.8], 1.5 M KCl, 1.5 M (NH_4_)_2_SO_4_, and 0.3 M MgSO_4_) was added to an RCA mixture containing a dNTP mixture (2.5 mM each), two primers (10 pmol), circular template–primer complex (50 ng), *Bst*-LF, and ThermoPol Buffer (20 mM Tris–HCl, 10 mM (NH_4_)_2_SO_4_, 10 mM KCl, 2 mM MgSO_4_, and 0.1% Triton X-100).

### Electrophoresis

RCA products were electrophoresed in 1.5% agarose gels and visualized using ethidium bromide. The electrophoresis buffer contained 222 mM Tris–borate buffer and 5 mM EDTA. The samples (5 μL) and a DNA marker (OneSTEP Marker 5, Nippon Gene) were electrophoresed for 30 min at 100 V. Gels were analyzed using an LAS-3000 Imaging System (Fujifilm) equipped with an ultraviolet filter (605DF40, Fujifilm).

### Fluorescence measurements

The intensity of SYBR Green I fluorescence emission was measured using a fluorescence plate reader (FluoroCount, Packard) with a 96-well white microwell plate (Thermo Scientific).

## Results

### Comparison of MW-RCA with conventional RCA using four DNA polymerases

**Fig 3:**
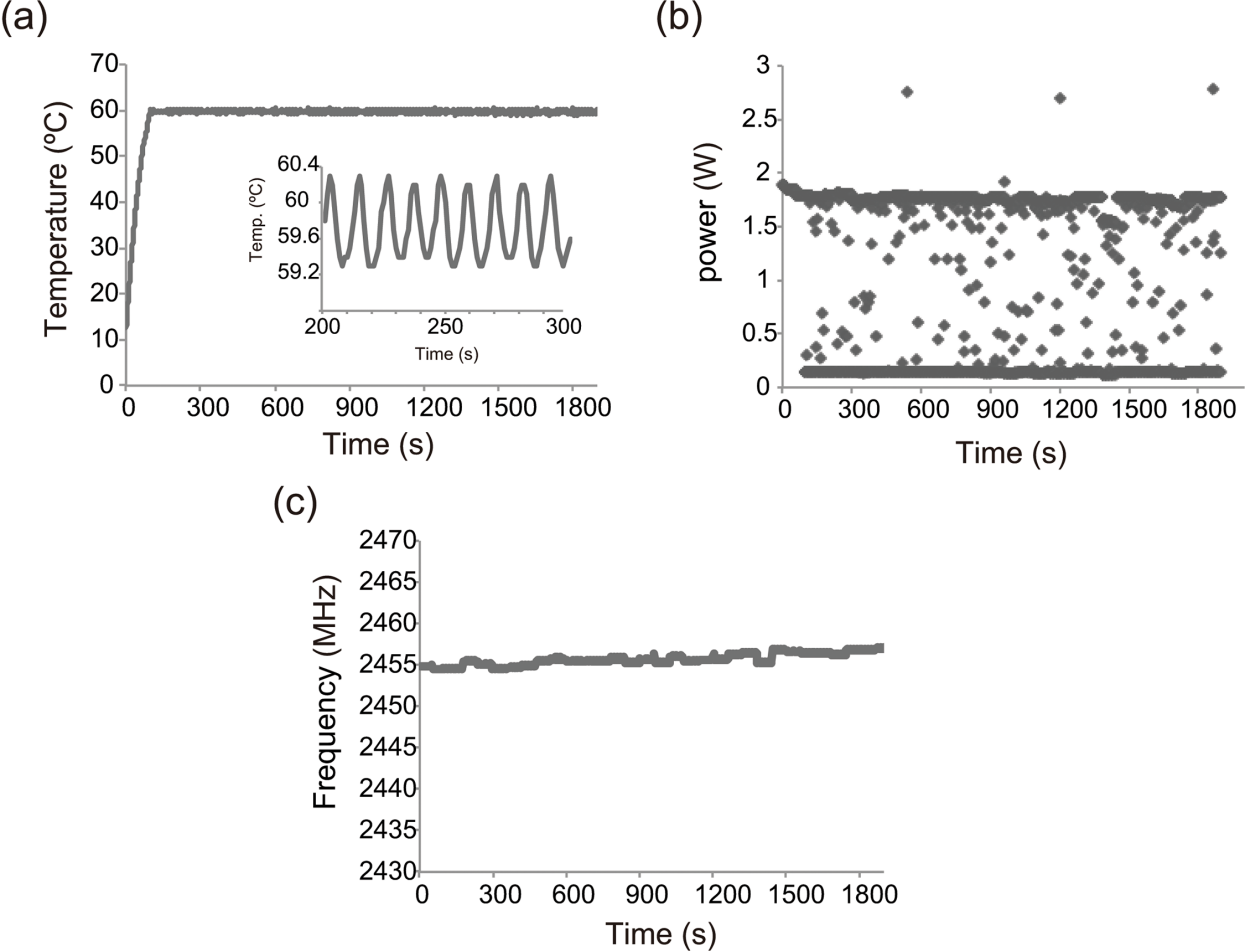
Temperature profile (a), electric power profile (b), and frequency profile (c) of MW-RCA using *Bst* DNA polymerase-LF at 60°C.

**Fig 4:**
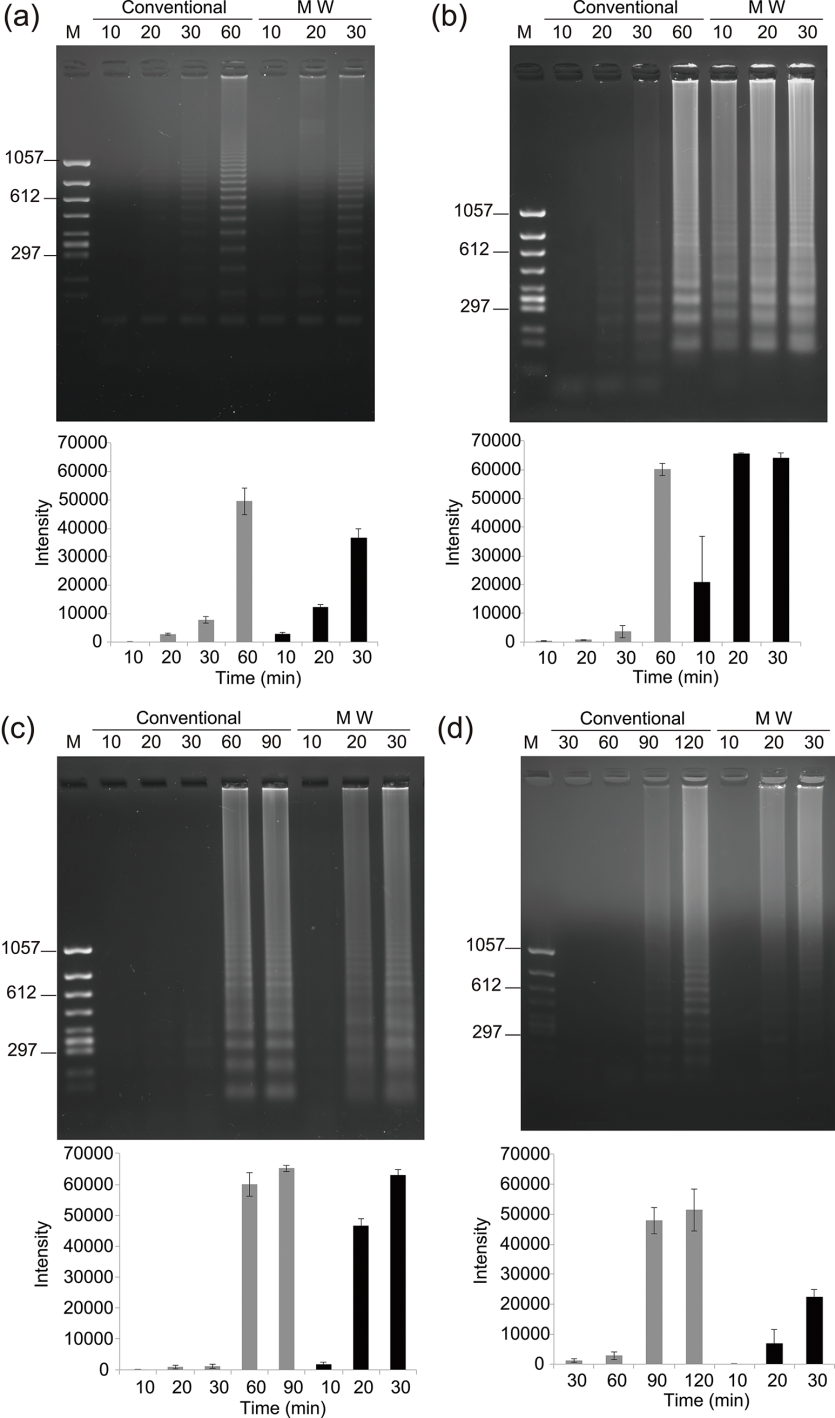
Electrophoresis and fluorescence intensity of DNA synthesized by four DNA polymerases under conventional and microwave heating. MW = microwave. (a) *Bst* DNA polymerase-LF, 10–60-min rolling circle amplification (RCA) and10–30-min microwave-assisted (MW)-RCA. (b) *Bst* DNA polymerase, 10–60-min RCA and 10–30-min MW-RCA (c) *Csa* DNA polymerase, 10–90-min RCA and 10–30-min MW-RCA. (d) 96–7 DNA polymerase, 10–120-min RCA and 10–30 min MW-RCA.

To determine the effect of microwave heating, MW-RCA was performed using four thermostable DNA polymerases that had strand displacement activity. Temperature, power, and frequency profiles of MW-RCA using *Bst*-LF at 30°C for 30 min is shown in Fig 3. A power profile of microwave is a value obtained by subtracting reflected power from incident power. Temperature profile data proved that MW-RCA was performed at precise temperatures. Conventional RCA and MW-RCA reactions were sampled at intervals from 10 to 60 min and 10 to 30 min, respectively, and analyzed using agarose gel electrophoresis and fluorescence emission (Fig 4). The use of the microwave applicator ended in 30 min under high temperature conditions.

A comparison of MW-RCA with conventional RCA using the *Bst*-LF revealed that the reaction product of MW-RCA was increased by a factor of four compared with conventional RCA (Fig 4A). Fluorescence intensity showed a 4-fold increase than conventional RCA in 30 min. Repetitive DNA sequences repeated four times (300 bp) and eight times (600 bp) were accurately replicated using MW-RCA using *Bst*-LF under microwave irradiation. The results of each assay showed that the yields of DNA produced by MW-RCA using the *Bst* DNA polymerase (60°C) were markedly greater compared with those by conventional RCA during the first 30 min (Fig 4B). The fluorescence intensities of the MW-RCA reaction sampled at 20 and 30 min and those of the conventional RCA reaction sampled at 60 min showed that the former plateaued at 20 min (Fig 4B). The *Bst* DNA polymerase was the most effective enzyme for MW-RCA. In the same way, MW-RCA using the *Csa* DNA polymerase was accelerated at 60°C compared with conventional RCA, and polymerization was increased at 20 min. In contrast, DNA synthesis was not detectable in the conventional RCA reaction at 30 min (Fig 4C). The fluorescence intensity in MW-RCA using *Csa* was higher by a factor of 30 at 30 min compared with that of the conventional RCA, and it was almost equal to that of conventional RCA at 60 and 90 min (Fig 4C). Reactions using the 96–7 DNA polymerase were incubated at 55°C, its optimum temperature, and showed an increase in discrete bands at 120 min, representing repeated sequences that were detected in the conventional RCA reaction. In contrast, MW-RCA using the 96–7 DNA polymerase showed diffuse migration of high molecular weight DNA molecules at 20 min, although DNA synthesis using conventional RCA was undetectable after 60 min. The fluorescence intensity of the conventional RCA reaction products at 90 min was twice as that of MW-RCA at 30 min (Fig 4D). Temperature and power profiles of four DNA polymerases under microwave heating are indicated in the supporting information (S1 File).

### Comparison of temperature profiles of RCA components using the microwave applicator and the heating block

**Fig 5:**
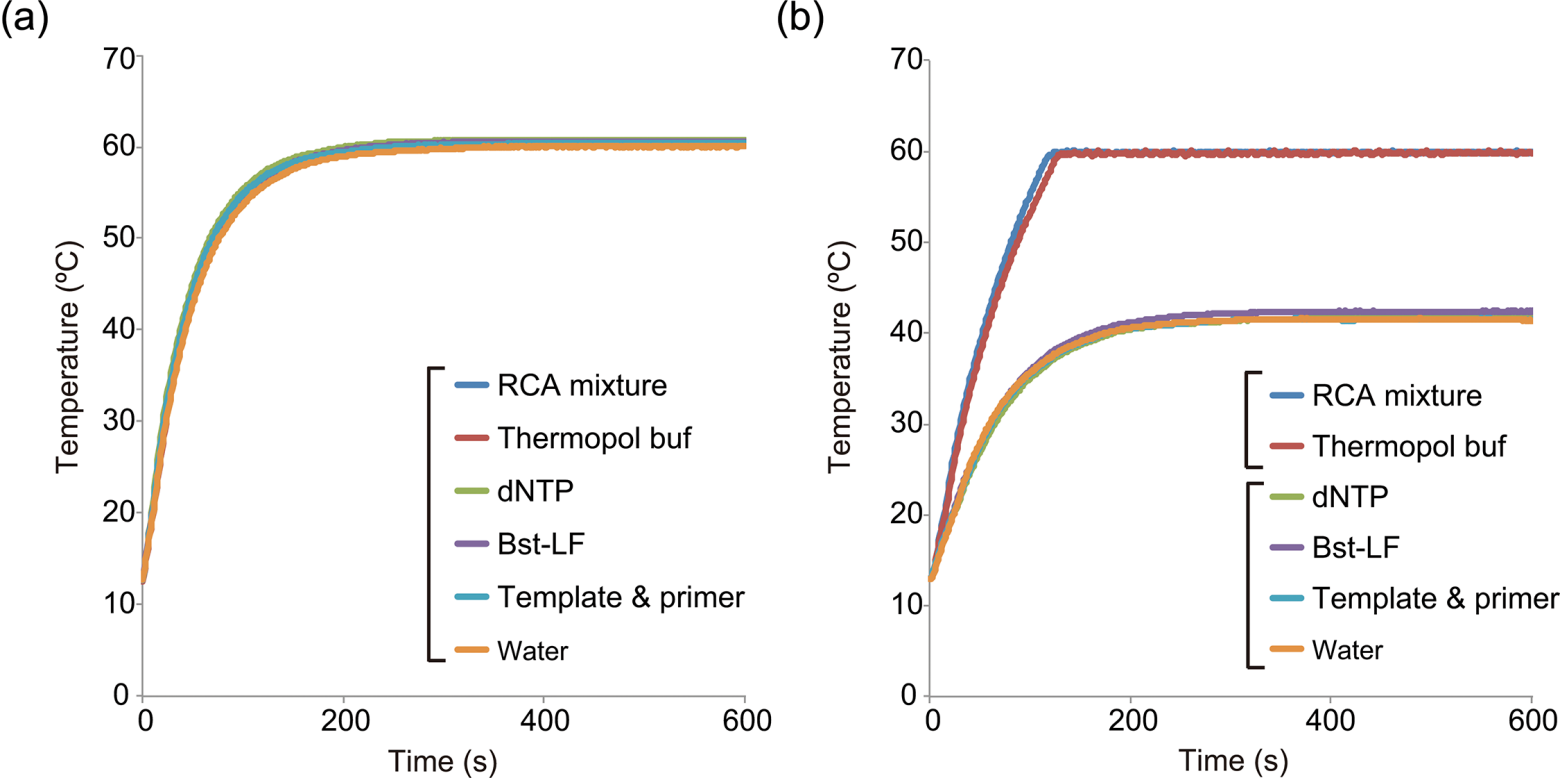
Comparison of temperatures of rolling circle amplification components from 13°C to 60°C using conventional (a) and microwave heating (b). Conventional heating was conducted in a 60°C heating block.

To determine the component of RCA by microwave selectivity heating, we measured the temperatures of the five components (circularized template with primers, dNTPs, ThermoPol Buffer, *Bst*-LF, and RNase-free water) of the RCA and MW-RCA mixtures for 10 min from 13°C to 60°C. A fiber-optic probe was used to measure the temperature of the conventional heating block. All six samples (RCA and its five components) attained 60°C using a 60°C heating block (Fig 5A). By microwave heating the RCA mixture including all components reached 60°C in 2 min. Moreover, the ThermoPol Buffer with 45 μL of RNase-free water reached 60°C by microwave heating 10 s after the addition of the RCA mixture. Four samples (dNTPs with 46 μL of RNase-free water, circularized template with primers in 50 μL of RNase-free water, 8 U of the *Bst* DNA polymerase in 50 μL of RNase-free water, and 50 μL of RNase-free water) reached a maximum temperature of 42°C by microwave heating in 10 min (Fig 5B). Profiles of microwave power are showed in supporting information (S3 Fig).

### Effect of microwave irradiation on the temperatures of higher concentrations of buffer components added to RCA reaction mixtures

**Fig 6:**
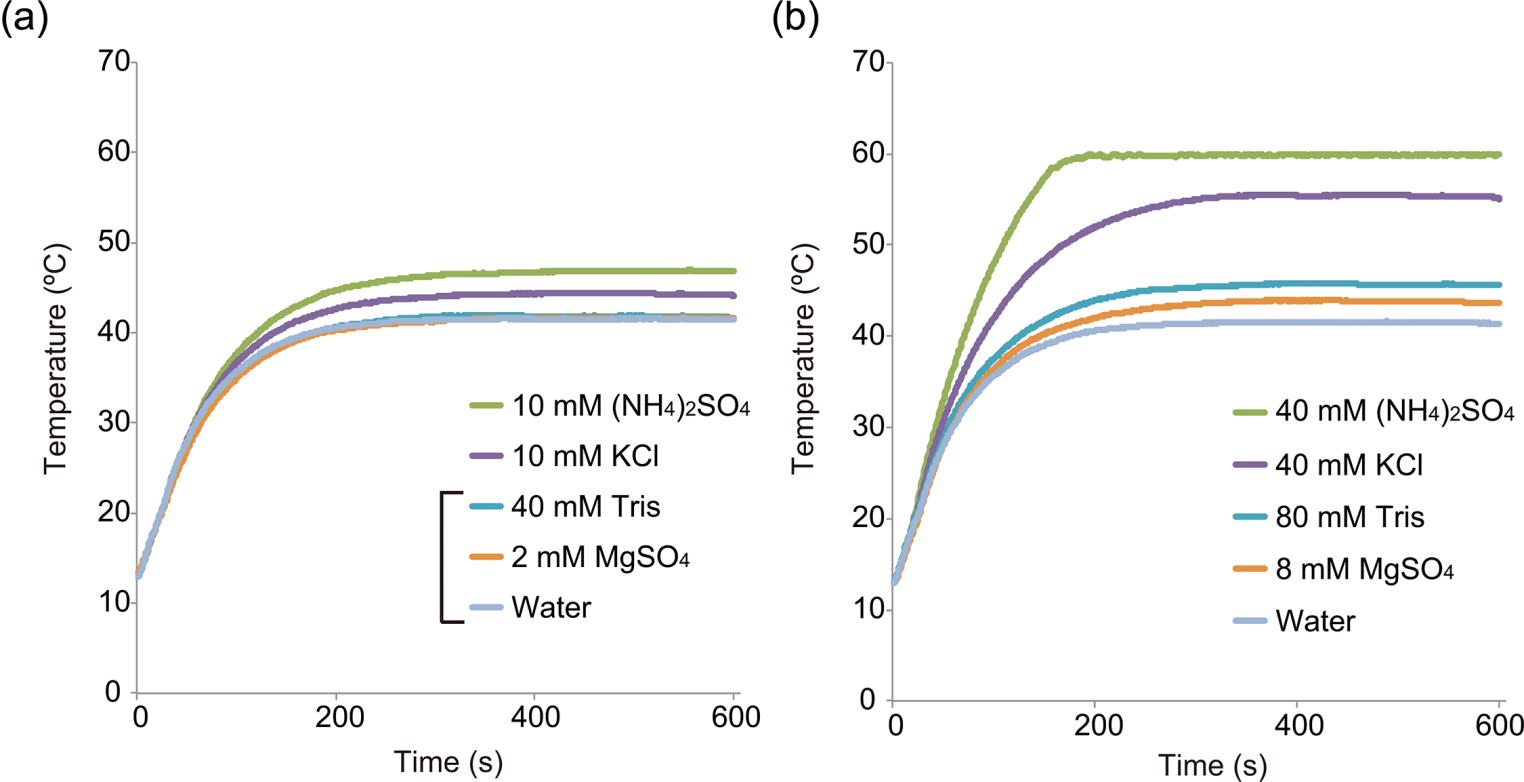
The temperatures of ThermoPol Buffer individual components at (a) 1-fold (b) and 4-fold higher concentrations heated from 13°C to 60°C by microwave heating.

The results shown in Fig 5 suggest that the ThermoPol Buffer was a primary factor leading to an increase in temperature under microwave irradiation. To determine whether the ThermoPol Buffer contained a specific thermal component that was selectivity affected by microwave irradiation, we measured the temperatures of each of the four ThermoPol Buffer components (Fig 6A). The microwave applicator heated the four components (20 mM Tris–HCl, 10 mM (NH_4_)_2_SO_4_, 10 mM KCl, and 2 mM MgSO_4_) from 13°C to 60°C for 10 min. The four components did not reach 60°C (Fig 6A), i.e., the temperature increase of 10 mM (NH_4_)_2_SO_4_ was the highest (47°C). In contrast, 10 mM KCl reached 44°C and the temperatures of 20 mM Tris–HCl and 2 mM MgSO_4_ (42°C) were nearly identical to the temperature of the RNase-free water.

Further, to examine the MW-RCA increased component of microwave selectivity heating, we measured the temperatures of 4-fold excess concentrations of buffer components under microwave irradiation (Fig 6B). The temperature of 40 mM (NH_4_)_2_SO_4_ reached 60°C after 195 seconds and that of 40 mM KCl plateaued at 55°C; the maximum temperature of 80 mM Tris–HCl was 46°C. Increasing the concentration of MgSO_4_ from 2 mM to 8 mM increased its maximum temperature from 42°C to 44°C. The quadruple density of all four samples produced an increase in temperature. Profiles of microwave power are indicated in the supporting information (S4 Fig).

### Comparison of RCA with MW-RCA reaction mixtures each containing a 4-fold increased concentration of each RCA component

**Fig 7:**
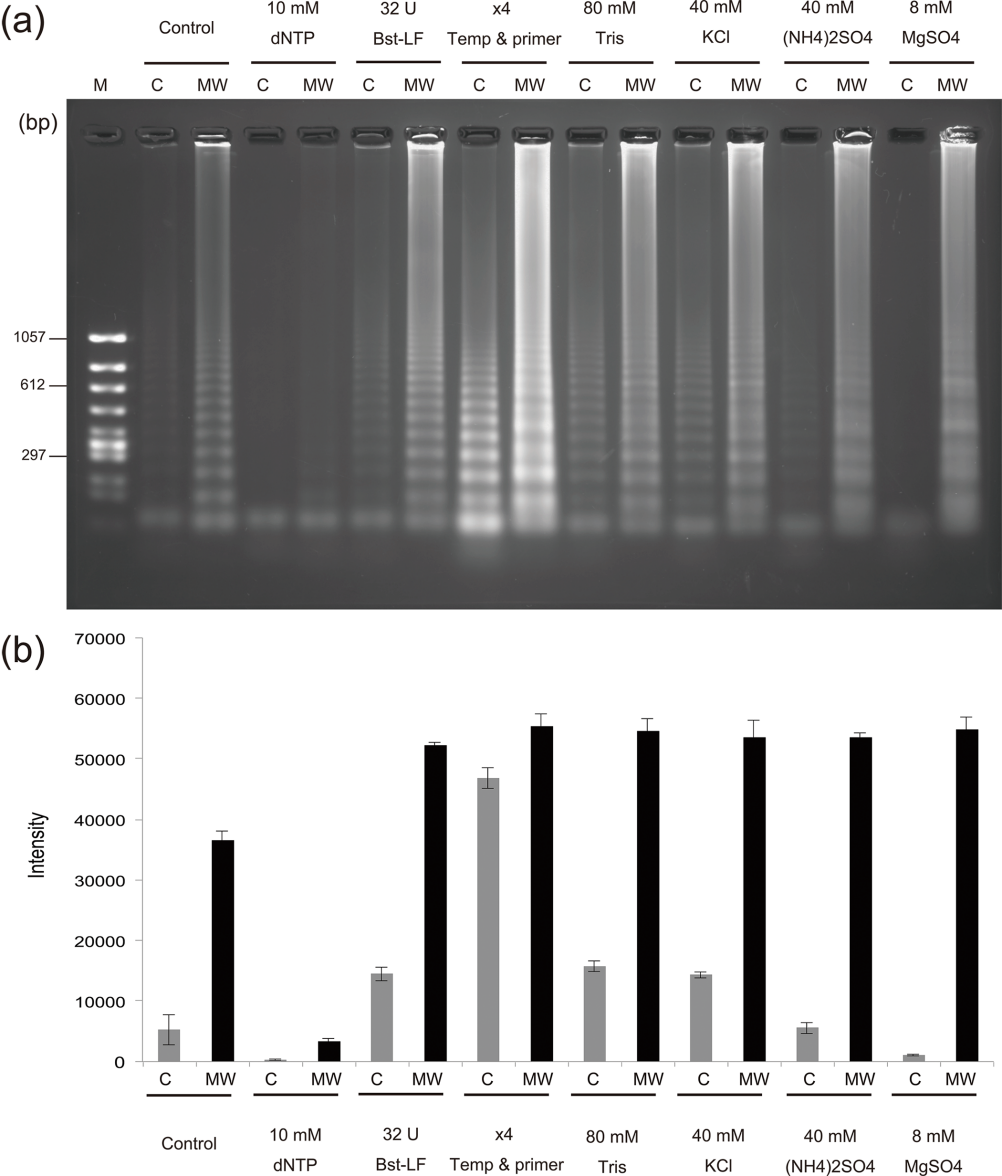
Microwave-assisted rolling circle amplification reaction mixtures containing 4-fold increased concentrations of one component. (a) Agarose gel electrophoresis results. (b) Fluorescence intensity of SYBR Green I. C = conventional, MW = microwave.

To reveal the effect of the selectivity heating in MW-RCA, we compared the efficiency of DNA amplification in the RCA and MW-RCA reactions mixtures containing a 4-fold excess concentration of each RCA component (dNTP, template–primers, *Bst*-LF, Tris–HCl, KCl, (NH_4_)_2_SO_4_, and MgSO_4_). All reactions were performed for 30 min at 60°C using a thermal cycler with the microwave applicator, and the reaction products were analyzed using agarose gel electrophoresis and SYBR Green assay. Profiles of temperature and power in microwave heating are indicated in the supporting information (S3 File). MW-RCA of the control and all seven types of MW-RCA reactions, each containing a 4-fold excess concentration of each RCA component, effectively amplified 75-bp circular templates compared with each control RCA (Fig 7). RCA reactions containing a 4-fold excess concentration of dNTPs incubated under conventional or microwave heating conditions did not amplify repetitive DNA (Fig 7A). Moreover, RCA reactions containing a 4-fold excess concentration of the *Bst*-LF were accelerated by conventional as well as microwave heating. RCA and MW-RCA reaction mixtures each containing template–primers at a 4-fold excess concentration were most effectively replicated by the other seven sample pairs (Fig 7A). The 4-fold increases in the concentrations of Tris–HCl and KCl accelerated RCA and MW-RCA reactions compared with controls. In contrast, MW-RCA reaction mixtures containing a 4-fold excess concentration of (NH_4_)_2_SO_4_ were accelerated to a greater extent compared with the control MW-RCA reaction, whereas the yield of the RCA reaction mixture containing a 4-fold excess concentration of (NH_4_)_2_SO_4_ was equal to that of the control conventional RCA reaction mixture. Similarly, MW-RCA using a 4-fold excess concentration of MgSO_4_ equivalently accelerated to an MW-RCA reaction containing a 4-fold excess concentration of (NH_4_)_2_SO_4_, although the conventional RCA reaction mixture containing a 4-fold excess concentration of MgSO_4_ yielded a slight increase of DNA.

## Discussion

Our research focused on the acceleration of RCA by microwave heating. To accomplish our objective, we developed a microwave applicator, which avoided the problem of temperature measurement under microwave irradiation and allowed us to implement new reaction conditions that enhanced conventional RCA. We considered the possibility that microwave-assisted enzymatic reactions are more effective at higher temperatures compared with those at typical physiological conditions. Using four thermostable DNA polymerases with strand displacement activity, we compared the products of MW-RCA and conventional RCA and found that microwave heating accelerated the reactions catalyzed by the four DNA polymerases compared with conventional heating at the same temperature (Fig 4). In a previous study, DNA amplification of MW-PCR, which was controlled at the precise temperature using the three steps, was reported [29]. In this study, RCA using four thermostable DNA polymerases was accelerated by microwave heating. A RCA reaction is equivalent to an elongation reaction of three steps (annealing, elongation, and denaturation) of PCR. These results of MW-RCA suggest that the reason of acceleration in MW-PCR was DNA polymerization by microwave heating.

Why was RCA with four heat-stable DNA polymerases accelerated by microwave heating, although the overall reaction temperatures were the same? In another report of microwave-assisted enzymatic reactions, the authors hypothesized that microwave effects on enzymatic digestion are due to the increased dipole moments of α-helices of proteins [33]. A microwave study using immobilized thermostable lipase B suggested that the effect of microwave irradiation of enzymes was due to superheating of the water layer near the enzyme surface [34]. A study using thermostable β-glucosidase under microwave irradiation at frequencies of 2.45 and 5.8 GHz suggested, with respect to the heating mechanism, that 5.8 GHz irradiation affected only the water molecules in the buffer solution, whereas 2.45 GHz acted on both water molecules and buffering ions [35].

To elucidate the mechanism of this microwave heating effect in RCA, we compared the temperature increase of RCA components under conventional or microwave heating (see Fig 5). The RCA components of the *Bst* DNA polymerase-LF reaction mixture were used for this purpose, because only the ThermoPol buffer of this polymerase is a nonproprietary reagent. The temperature increases for all RCA components, which were placed in a 60°C heating block, were the same. Microwave heating to 60°C increased the temperature of the RCA mixture as well as that of the ThermoPol buffer to 60°C. In contrast, the temperature of the four RCA components (*Bst* DNA polymerase-LF, dNTP, template–primer, and water) was 42°C. These data indicate that the ThermoPol buffer in MW-RCA was selectively heated by microwave irradiation. ThermoPol buffer, which contains a high concentration of ionic molecules, is considered to be heated by conduction loss of microwave irradiation [19, 20].

We postulated that the acceleration of MW-RCA is caused by the components of Thermopol buffer on selective microwave heating. The approach to the microwave heating effect is expected to change the amount of the ThermoPol buffer, which was selectively heated by microwave irradiation. However, a substantial change in concentration of the ThermoPol buffer is impossible because enzymatic reactions have an optimum concentration. We focused on the four components of ThermoPol buffer. We hypothesized that the microwave selective heating could be observed with heating of only one of each buffer component under microwave irradiation. We accordingly prepared four samples of one-fold ThermoPol buffer components [Tris-HCl, KCl, (NH_4_)_2_SO_4_, and MgSO_4_] and four samples of ThermoPol buffer components in four-fold excess concentrations, and measured their temperatures under microwave heating (Fig 6). Temperature measurements of ThermoPol buffer components at four-fold excess concentrations showed an increase in temperature compared with one-fold concentrations. The microwave heating method correlated with the increase in concentrations of buffer components.

We performed MW-RCA reactions containing a four-fold higher concentration of each RCA component [dNTP, template–primers, *Bst* DNA polymerase-LF, Tris-HCl, KCl, (NH_4_)_2_SO_4_, and MgSO_4_] to identify a link between microwave selective heating and DNA amplification. There was no influence of pH change by the additional buffer component in RCA (S4 File). All results of microwave heating were accelerated compared with conventional heating (Fig 7). Two notable results were observed. MW-RCA with 40 mM of (NH_4_)_2_SO_4_ was accelerated relative to the control MW-RCA, whereas RCA with 40 mM of (NH_4_)_2_SO_4_ was amplified like the control of conventional RCA. (NH4)_2_SO_4_ plays the role of a buffering solution and is not important for DNA polymerization. In contrast, Mg^2+^ is necessary for the function of DNA polymerase. The conventional RCA reaction mixture containing 8 mM of MgSO_4_ contained a slightly amplified concentration of DNA. Conventional RCA with 8 mM of MgSO_4_ was not at optimum concentration, although MW-RCA reaction in mixtures containing 8 mM of MgSO_4_ was accelerated. As shown in Figs 5 and 6, buffer solutions of (NH_4_)_2_SO_4_ and MgSO_4_ showed large temperature increases per 1 mM under microwave irradiation. These findings suggest a link between the amplification of MW-RCA and the temperature increase of (NH_4_)_2_SO_4_ and MgSO_4_ under microwave irradiation.

The effect of Tris-HCl and KCl on MW-RCA was unclear, given that the conventional results of RCA containing four-fold excess concentrations of Tris-HCl (80 mM) and KCl (40 mM) were effectively amplified relative to control RCA (Fig 7B). Control RCA may improve the reaction by buffer composition of Tris-HCl and KCl. The complex dielectric constant of protein in water or buffer solution has been described in [35, 36]. We need to measure the complex dielectric constant of RCA components to understand the microwave heating effect of MW-RCA.

In conclusion, we report here the development of a microwave applicator for enzymatic reactions and demonstrate the effect of microwave heating on RCA of DNA. The temperature profiles of RCA components by microwave heating suggest that the ionic components of the ThermoPol buffer are selectively heated and that the rate of temperature increase induced by microwave heating depends on the components of a molecule and the concentrations of buffer components. These findings indicate that (NH_4_)_2_SO_4_ and MgSO_4_ play important roles in the mechanism of MW-RCA. The shortened reaction time and higher product yields indicate that microwave heating systems can be widely used to enhance enzymatic reactions. Studies are being conducted to further improve the maximum incubation time at temperatures higher than 60°C as well as to enhance the visualization of the heating process. Preliminary results indicate that MW-RCA can be accelerated at 37°C.

## Supporting Information

S1 Fig Temperature profiles of MW-RCA (Fig 4) using for DNA polymerization. (a) *Bst* DNA polymerase-LF, 10–30-min MW-RCA. (b) *Bst* DNA polymerase, 10–30-min MW-RCA. (c) *Csa* DNA polymerase, 10–30-min MW-RCA. (d) 96–7 DNA polymerase, 10–30-min MW-RCA.
(EPS)

S2 Fig Electric power profiles of MW-RCA (Fig 4) using for DNA polymerization. (a) *Bst* DNA polymerase-LF, 10–30-min MW-RCA. (b) *Bst* DNA polymerase, 10–30-min MW-RCA. (c) *Csa* DNA polymerase, 10–30-min MW-RCA. (d) 96–7 DNA polymerase, 10–30-min MW-RCA.
(EPS)

S3 Fig Electric power profiles of RCA components (Fig 5B) from 13°C to 60°C. (a) RCA mixture (b) ThermoPol Buffer (c) dNTP (d) *Bst* DNA polymerase-LF (e) Template & primer (f) water
(EPS)

S4 Fig Electric power profiles of ThermoPol Buffer components (Fig 6) of x1 and x4 from 13°C to 60°C. (a) 10 mM (NH_4_)_2_SO_4_ (b) 40 mM (NH_4_)_2_SO_4_ (c) 10 mM KCl (d) 40 mM KCl (e) 20 mM Tris–HCl (f) 80 mM Tris–HCl (g) 2 mM MgSO_4_ (h) 8 mM MgSO_4_.
(EPS)

S5 Fig Temperature profiles of MW-RCA reaction mixtures (Fig 7) from each containing a 4-fold increased concentration of each RCA component from 13°C to 60°C. (a) Control_MW-RCA (b) 10 mM dNTP_MW-RCA (c) 32 U *Bst* DNA polymerase-LF_MW-RCA (d) ×4 Template & primer_MW-RCA (e) 80 mM Tris–HCl_MW-RCA (f) 40 mM KCl_MW-RCA (g) 40 mM (NH_4_)_2_SO_4__MW-RCA (h) 8 mM MgSO_4__MW-RCA.
(EPS)

S6 Fig Electric power profiles of MW-RCA reaction mixtures (Fig 7) each containing a 4-fold increased concentration of each RCA component at 60°C. (a) Control_MW-RCA (b) 10 mM dNTP_MW-RCA (c) 32 U *Bst* DNA polymerase-LF_MW-RCA (d) ×4 Template & primer_MW-RCA (e) 80 mM Tris–HCl_MW-RCA (f) 40 mM KCl_MW-RCA (g) 40 mM (NH_4_)_2_SO_4__MW-RCA (h) 8 mM MgSO_4__MW-RCA.
(EPS)

S7 Fig Comparison of NEB buffer with the buffer prepared by us in RCA using *Bst* DNA polymerase-LF. 1: NEB Thermopol-buffer (pH 8.8), 2: Prepared Thermopol-buffer (pH 8.80), 3: Prepared Thermopol-buffer (pH 8.34).
(EPS)

S1 File Temperature and power profiles of MW-RCA (Fig 4). S1 File shows the profiles of temperature (S1 Fig) and power (S2 Fig) to support the results of Fig 4.
(DOCX)

S2 File Power profiles of microwave heating experiments of Figs 5 and 6. S3 Fig shows the power profiles of RCA components, which were heated to reach the 60°C by microwave. S4 Fig also shows the power profiles of ThermoPol Buffer components with each concentration.
(DOCX)

S3 File Temperature and power profiles of MW-RCA (Fig 7). S3 File shows the profiles of temperature (S5 Fig) and power (S6 Fig) to support the results of Fig 7.
(DOCX)

S4 File Comparison of ThermoPol Buffer (NEB) with prepared buffer for RCA. The 1× Thermopol Reaction Buffer [20 mM Tris–HCl, 10 mM (NH4)_2_SO_4_, 10 mM KCl, 2 mM MgSO_4_, 0.1% Triton X-100] was prepared at pH 8.8. The component of *Bst* DNA polymerase-LF [10 mM Tris–HCl pH 7.5, 50 mM KCl, 0.1 mM EDTA, 1 mM DTT, 0.1% Triton X-100, 50% glycerol] was prepared. The pH of Scale-up fictitious RCA mixture containing Thermopol-buffer and enzyme component was measured (pH 8.71). The pH of fictitious RCA mixtures of one 4-fold constituent of Thermopol-buffer were measured (4-fold Tris–HCl: 8.73, 4-fold KCl; 8.69, 4-fold (NH4)_2_SO_4_: 8.37, 4-fold MgSO_4_: 8.67). Thus, to assess the effect of pH, RCA using the Thermopol-buffer prepared by us (pH 8.80 and 8.34) was performed and was compared with a control RCA using NEB Thermopol-buffer. As a result, no effect of RCA products by pH alteration of this range was confirmed by electrophoresis and fluorescence analysis.
(DOCX)
