# Supplementary material for: Controlled Microwave Heating Accelerates Rolling Circle Amplification
Source: PLoS One. 2015 Sep 8;10(9):e0136532. doi: 10.1371/journal.pone.0136532 (PMC4562646; doi:10.1371/journal.pone.0136532)
Supplement: S1 File — shows the profiles of temperature (S1 Fig) and power (S2 Fig) to support the results of Fig 4. (DOCX) [file pone.0136532.s008.docx]

**S1 File. Temperature and power profiles of MW-RCA (Fig 4).**  S1 File shows the profiles of temperature (S1 Fig) and power (S2 Fig) to support the results of Fig 4.

S1 File shows the profiles of temperature (S1 Fig) and power (S2 Fig) to support the results of Fig 4.
