# Supplementary material for: Controlled Microwave Heating Accelerates Rolling Circle Amplification
Source: PLoS One. 2015 Sep 8;10(9):e0136532. doi: 10.1371/journal.pone.0136532 (PMC4562646; doi:10.1371/journal.pone.0136532)
Supplement: S2 File — S3 Fig shows the power profiles of RCA components, which were heated to reach the 60°C by microwave. S4 Fig also shows the power profiles of ThermoPol Buffer components with each concentration. (DOCX) [file pone.0136532.s009.docx]

**S2 File. Power profiles of microwave heating experiments of Fig 5 and 6.** S3 Fig shows the power profiles of RCA components, which were heated to reach the 60°C by microwave. S4 Fig also shows the power profiles of ThermoPol Buffer components with each concentration.
