# Supplementary material for: Controlled Microwave Heating Accelerates Rolling Circle Amplification
Source: PLoS One. 2015 Sep 8;10(9):e0136532. doi: 10.1371/journal.pone.0136532 (PMC4562646; doi:10.1371/journal.pone.0136532)
Supplement: S3 File — shows the profiles of temperature (S5 Fig) and power (S6 Fig) to support the results of Fig 7. (DOCX) [file pone.0136532.s010.docx]

**S3 File. Temperature and power profiles of MW-RCA (Fig 7).**  S3 File shows the profiles of temperature (S5 Fig) and power (S6 Fig) to support the results of Fig 7.
