# Supplementary material for: Controlled Microwave Heating Accelerates Rolling Circle Amplification
Source: PLoS One. 2015 Sep 8;10(9):e0136532. doi: 10.1371/journal.pone.0136532 (PMC4562646; doi:10.1371/journal.pone.0136532)
Supplement: S4 File — The 1× Thermopol Reaction Buffer [20 mM Tris–HCl, 10 mM (NH4)2SO4, 10 mM KCl, 2 mM MgSO4, 0.1% Triton X-100] was prepared at pH 8.8. The component of Bst DNA polymerase-LF [10 mM Tris–HCl pH 7.5, 50 mM KCl, 0.1 mM EDTA, 1 mM DTT, 0.1% Triton X-100, 50% glycerol] was prepared. The pH of Scale-up fictitious RCA mixture containing Thermopol-buffer and enzyme component was measured (pH 8.71). The pH of fictitious RCA mixtures of one 4-fold constituent of Thermopol-buffer were measured (4-fold Tris–HCl: 8.73, 4-fold KCl; 8.69, 4-fold (NH4)2SO4: 8.37, 4-fold MgSO4: 8.67). Thus, to assess the effect of pH, RCA using the Thermopol-buffer prepared by us (pH 8.80 and 8.34) was performed and was compared with a control RCA using NEB Thermopol-buffer. As a result, no effect of RCA products by pH alteration of this range was confirmed by electrophoresis and fluorescence analysis. (DOCX) [file pone.0136532.s011.docx]

**S4 File. Comparison of ThermoPol Buffer (NEB) with prepared buffer to RCA.**

The 1× Thermopol Reaction Buffer [20 mM Tris–HCl, 10 mM (NH4)_2_SO_4_, 10 mM KCl, 2 mM MgSO_4_, 0.1% Triton X-100] was prepared at pH 8.8. The component of *Bst* DNA polymerase-LF [10 mM Tris–HCl pH 7.5, 50 mM KCl, 0.1 mM EDTA, 1 mM DTT, 0.1% Triton X-100, 50% glycerol] was prepared. The pH of Scale-up fictitious RCA mixture containing Thermopol-buffer and enzyme component was measured (pH 8.71). The pH of fictitious RCA mixtures of one 4-fold constituent of Thermopol-buffer were measured (4-fold Tris–HCl: 8.73, 4-fold KCl; 8.69, 4-fold (NH4)_2_SO_4_: 8.37, 4-fold MgSO_4_: 8.67). Thus, to assess the effect of pH, RCA using the Thermopol-buffer prepared by us (pH 8.80 and 8.34) was performed and was compared with a control RCA using NEB Thermopol-buffer. As a result, no effect of RCA products by pH alteration of this range was confirmed by electrophoresis and fluorescence analysis.
